# Colchicine Is a Weapon for Managing the Heart Disease Among Interstitial Lung Disease With Viral Infection: Have We Found the Holy Grail?

**DOI:** 10.3389/fcvm.2022.925211

**Published:** 2022-06-28

**Authors:** Jun-Jun Yeh, Tuey-Wen Hung, Cheng-Li Lin, Tsung-Tse Chen, Pei-Xuan Liw, Ya-Lun Yu, Chia-Hung Kao

**Affiliations:** ^1^Department of Family Medicine, Geriatric Medicine, Chest Medicine and Medical Research, Ditmanson Medical Foundation Chia-Yi Christian Hospital, Chiayi, Taiwan; ^2^College of Medicine, China Medical University, Taichung, Taiwan; ^3^Department of Family Medicine and Geriatric Medicine, Ditmanson Medical Foundation Chia-Yi Christian Hospital, Chiayi, Taiwan; ^4^Management Office for Health Data, China Medical University Hospital, Taichung, Taiwan; ^5^Graduate Institute of Biomedical Sciences, College of Medicine, China Medical University, Taichung, Taiwan; ^6^Department of Nuclear Medicine and Positron Emission Tomography (PET) Center, China Medical University Hospital, Taichung, Taiwan; ^7^Department of Bioinformatics and Medical Engineering, Asia University, Taichung, Taiwan; ^8^Center of Augmented Intelligence in Healthcare, China Medical University Hospital, Taichung, Taiwan

**Keywords:** colchicine, pericarditis, endocarditis, myocarditis, cardiomyopathy

## Abstract

**Background:**

This study investigated the effect of colchicine use on the risks of heart disease (HD), pericarditis, endocarditis, myocarditis, cardiomyopathy, cardiac arrhythmia, and cardiac failure in patients having interstitial lung disease (ILD) with virus infection (ILD cohort).

**Methods:**

We retrospectively enrolled ILD cohort between 2000 and 2013 from the Longitudinal Health Insurance Database and divided them into colchicine users (*n* = 12,253) and colchicine non-users (*n* = 12,253) through propensity score matching. The event of interest was the diagnosis of HD. The incidence of HD was analyzed using multivariate Cox proportional hazards models between colchicine users and the comparison cohort after adjustment for age, sex, medication, comorbidities, and index date based on the time-dependent analysis.

**Results:**

Colchicine users had a significantly lower risk of HD (*aHR* = 0.87, 95% confidence interval (CI]) = 0.82–0.92) than did the colchicine non-user. For colchicine non-users as the reference, the *aHR* (95% CI) of the patients who received colchicine of 2–7, 8–30, 31–150, and > 150 days were 0.89 (0.81–0.98), 0.84 (0.76–0.94), 090 (0.80–0.99), and 0.83 (0.74–0.93), respectively; regardless of duration use, the lower risk of HD persisted in colchicine users. The cumulative incidence of HD in colchicine users was significantly lower than that in the colchicine non-users (log-rank *p* < 0.001).

**Conclusion:**

The addition of short-term or long-term colchicine to standard medical therapy may have benefits to prevent the HD among the ILD patients concurrent with a virus infection or comorbidities even in elderly patients.

## Introduction

Cardiac and pulmonary impairment is a late manifestation of the interstitial lung disease (ILD). The ILD including the autoimmune related—sarcoidosis, connective tissue disease (CTD), idiopathic pulmonary fibrosis (IPF), and occupation lung disease. The late course of CTD-lupus associated with primary heart disease (HD)- myocarditis, pericarditis, endocarditis, cardiac arrhythmia, cardiomyopathy, and heart failure (1). Patients with viral infection can be an acute, subacute, or chronic disorder and may present with focal or diffuse involvement of the myocardium can develop temporary or permanent impairment of cardiac function including acute cardiomyopathy with hemodynamic compromise or severe cardiac arrhythmia.

The inflammasomes such as nod-like receptor family pyrin domain containing 3 (NLRP3) inflammasome play a critical role for the primary exacerbation of the ILD. For example, the activation of the NLRP3 inflammasome by integrating multiple cellular and molecular signaling implicates robust fibroblast proliferation with activation of myofibroblast, matrix deposition, and aberrant epithelial–mesenchymal function. And, the NLRP3 inflammasome may trigger the release of the proinflammatory cytokines such as interleukin (IL)-1β, IL-6, and IL-18 and initiate or exacerbate the HD (2, 3). The rate of virus infection in the ILD-IPF is high (4, 5). If the ILD-IPF concurrent with viral infection, the combination of the crosstalk of the NLRP3 inflammasome and viruses can enhance immune responses with inflammasome-associated molecules in the development, progression, and exacerbation of ILD-IPF, leading to the higher risk of HD (6, 7). Altogether, the ILD-IPF, CTD, autoimmune disease, viral infection, and HD may coexist and interplay each other.

The role of colchicine in the management of ILD-IPF is inconclusive (8). However, in the Coronavirus disease 2019 (COVID-19) era, the colchicine may play an auxiliary role in the treatment of the coronavirus, especially with comorbidities such as hypertension, hyperlipidemia, and diabetes in ongoing study (9). Colchicine mainly acts on two types of immune system cells, namely neutrophils and macrophages, and reduces the production of proinflammatory cytokines, such as IL-1β and IL-18, thus lowering the levels of IL-6 and tumor necrosis factor-alpha. The ability of colchicine to modulate NLRP3 inflammasome might represent a therapeutic strategy for virus-related HDs among the ILD-CTD (10, 11). No study has investigated the effect of colchicine on the risk of HD in patients with ILD concurrent with viral infection. Therefore, we address this topic by analyzing the general population.

## Materials and Methods

### Data Sources

Our data for this population-based retrospective cohort study were obtained from the Longitudinal Health Insurance Database (LHID 2000), a subset database of the National Health Insurance Research Database (NHIRD) which contains a randomly sampled representative database of 1 million participants from the registry of all beneficiaries in 2000. The NHIRD is managed by Taiwan’s National Health Research Institutes and contains registration files and claims data for reimbursement and information regarding medical visits including outpatient emergency department and hospitalization. Because the database contains deidentified secondary data that are provided to the public for research purposes, informed consent is not required. All the diagnostic codes of the claims are recorded according to the *International Classification of Diseases, Ninth Revision, Clinical Modification* (*ICD-9-CM*). This study was approved by the Research Ethics Committee of China Medical University Hospital (CMUH104-REC2-115-AR-4).

### Patient Enrollment and Sample Collection

**FIGURE 1:**
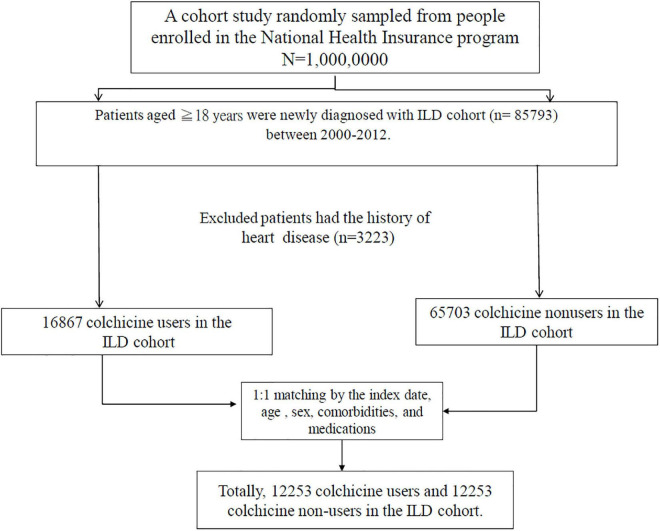
Flow chart.

**FIGURE 2:**
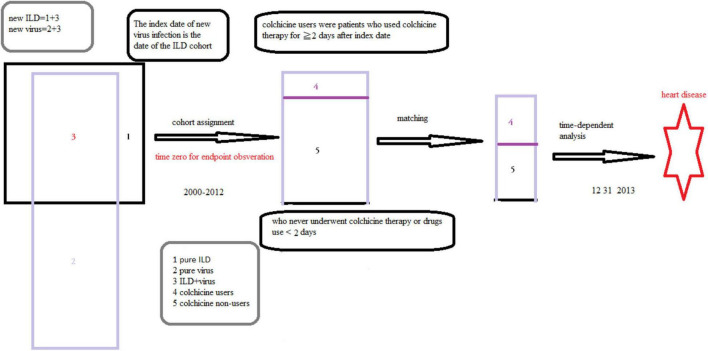
The selection of patients and definition of the study cohort and speculation of this study.

Initially, we identified patients with ILD from the NHIRD. For this study, patients with two or more outpatient visits or one hospitalization for ILD (new ILD, *ICD-9-CM* codes 135, 237.7, 272.2, 277.3, 277.8, 500–505, 506.4, 508.1, 508.8, 515–516, 446.21, 446.4, 495, 517.2, 517.8, 518.3, 555, 710, 714.81, 720, and 759.5) between 2000 and 2012 were entered into the study. The diagnosis of a new viral infection (*ICD-9-CM* codes 0.42, 0.53, 070.20, 070.22, 070.30, 070.32, 070.41, 070.44, 070.51, 070.54, 0.75, 0.78.5, 079.0–79.6, 079.81–079.83, 079.88–079.89, 480, and 486–488) were identified (12–14). Patients aged ≥ 18 years with ILD and viral infection were included in the ILD cohort. The index date of the new viral infection was the date of the ILD cohort. Patients who were aged < 18 years and had a history of HD before entry into the study were excluded (Figures 1, 2). Details regarding *ICD-9-CM* codes are listed in Supplementary Table 1.

Yang et al. reported that patients with IPF had concurrent pneumonia. Cytomegalovirus infection, influenza, and respiratory failure were the main causes for admission based on information provided in the NHIRD (12, 13, 15). Meanwhile, viral infections such as human immunodeficiency virus, herpes virus, and hepatitis B have been identified as predisposing factors for ILD and viral myocarditis was associated with cardiac arrhythmia, and cardiac failure (15–17). Therein, viral infection and ILD coexist and interplay, leading to HD. These studies support our speculations (Figures 1, 2).

### Colchicine Users Without Gout Subcohort

Colchicine users who had ILD with gout. The non-gout conditions such as sarcoidosis, Behcet’s syndrome, autoimmune diseases, and hypertension-related diseases (18) were included in the colchicine users without gout subcohort (Supplementary Table 1; *ICD-9-CM* codes).

### The Comorbidities and Medications in Place of the Laboratory Data

The severity of ILD could be evaluated based on the system inflammatory marker C-Reactive protein (CRP), interleukin (IL), glucose, hypoxemia status—the ratio of arterial oxygen partial pressure (PaO_2_ in mmHg) to fractional inspired oxygen (FiO_2_), sugar level, or blood pressure level (systolic blood pressure, sBP, diastolic pressure, dBP, mean blood pressure, and mBP). These clinical data were unavailable in NHIRD. However, the severity of ILD is correlated well with Charlson Comorbidity Index (CCI) (19). In this study, the components of the comorbidities and medications were similar to CCI. Meanwhile, we could consider the diabetes, hypertension, hyperlipidemia, gout as the tumor-related diseases and chronic liver disease as the system inflammatory disease (in proxy to CRP and IL), the COPD, pulmonary embolism (in proxy to PaO2/FiO2) as the respiratory insufficiency, the hypertension, chronic kidney disease (CKD), stroke (in proxy to sBP, dBP, and mBP) as the hypertension-related diseases (20) (Supplementary Table 2 and Supplementary Figure 1).

Altogether, the ILD-related complications were the major determinant of hospital mortality rather than ILD *per se*, CRP, IL, glucose, sBP, dBP, and mBP. In this time—dependent study, the age, sex, developing comorbidities, and medications of ILD were the accurate predictors of HD (19, 21, 22). Therefore, comorbidities, concurrent medications, and diseases were included in the analyses instead of clinical data to evaluate the effects of these clinical factors on HD in ILD (20, 21, 23) (Supplementary Table 2 and Supplementary Figure 1).

In the recent Tseng et al. study, they found that chest tightness (CAD, venous thrombosis), clubbing finger (COPD, pulmonary embolism), and frequency of admission after medications (stroke, CAD) were predictors of the severity of the ILD (IPF) (24). In similar scenario, the durations of the medications, frequency of medical services enter into this study. We found that the comorbidity (*n*> 1) in parallel with higher rate of the medications and longer durations (> 150 days), contributing to lower *aHR* for HD (Table 1). However, these postulations warrant further study.

**TABLE 1:** Incidence and hazards ratio (HR) of heart disease (HD) measured by age, sex, and comorbidity in colchicine users compared with those in colchicine non-users by Cox proportional hazard models with time-dependent exposure covariates.

	No matched					Propensity score matched				
			
	Colchicine					Colchicine				
			
	No (*N* = 65,073)		Yes (*N* = 16,867)			No (*N* = 12,253)		Yes (*N* = 12,253)		
					
Variables	Event	Rate^#^	Event	Rate^#^	Adjusted HR† (95% CI)	Event	Rate^#^	Event	Rate^#^	Adjusted HR† (95% CI)
**Age, years**										
18–49	3,316	12.8	688	11.1	0.79 (0.71, 0.87)***	555	13.1	513	11.6	0.83(0.74, 0.94)***
50–64	3,677	28.0	980	24.3	0.86 (0.80, 0.94)***	876	27.6	720	23.2	0.82 (0.74, 0.90)***
※65 +	2,849	55.4	887	47.6	0.86 (0.79, 0.94)***	670	52.2	600	45.0	0.93 (0.83, 1.04)
**Sex**										
Female	6,017	23.4	914	23.2	0.81 (0.75, 0.88)***	1,038	25.9	826	22.2	0.84 (0.77, 0.92)***
Male	3,825	20.6	1,641	20.1	0.90 (0.83, 0.96)***	1,063	22.7	1,007	19.6	0.92 (0.82, 0.98)*
**Comorbidity**										
No	815	8.00	43	6.67	0.83 (0.61, 1.12)	45	7.12	43	6.67	0.85 (0.56, 1.29)
Yes♥	9,027	26.5	2,512	22.0	0.88 (0.84, 0.92)***	2,056	25.5	1,790	21.8	0.85 (0.80, 0.91)***

*Rate#, incidence rate, per 1,000 person-years; Adjusted HR†: multivariable analysis including age, sex, comorbidities, and medications.*

*p < 0.05; ***P < 0.001.

*※The old age with higher drug adverse reaction contributing to lower drug adherence in parallel with colchicine having null effect on the aHR for HD.*

*♥The frequency of medical visit in the comorbidity (n > 1) was high, the higher frequency of medical services contributing to the higher drug adherence for anti-hypertension (90% with high adherence PDC≧80%) in parallel with colchicine having lower aHR for HD.*

### The Fluctuation of Sugar Blood Pressure and Uric Acid in Relationship to the Heart Disease

In the biochemistry standpoints, the fluctuation of sugar blood pressure and uric acid triggers the NLRP3 inflammasome or IL6 activity, leading to HD. Xu et al. that the high or low uric acid were predictive of the HD such as heart failure (not the uric acid per sec) (25). Meanwhile, the fluctuation of fasting blood glucose (diabetes) and total cholesterol levels (hyperlipidemia), sBP (hypertension), and body mass index (gout) was an independent predictor of HD (not the BP, sugar per sec) (26, 27). In this study, the most important factors for HD such as hypertension (62.4%), hyperlipidemia (77.7%), diabetes, gout (58.54%), smoking (COPD), lifestyle (100%, colchicine use) entered into analysis. Perhaps, these policies may avoid the bias for without the laboratory data.

### Case Definitions

Because acute biological effects of colchicine require 24–48 h to fully develop (10), we defined the case cohort (colchicine users) as patients who used colchicine therapy for ≥ 2 days after the index date. Patients who never underwent colchicine therapy or used drugs for < 2 days were included as controls (colchicine non-users) in the present study, and they were randomly assigned index dates between 2000 and 2012.

Meanwhile, the colchicine level, the level of renal and liver function was unavailable in NHILD. We replace the colchicine level with the duration of colchicine use, at least 2-day use, the liver function with the liver disease such as the liver cirrhosis, the renal function with the chronic kidney disease.

The date of the first ILD cohort was defined as the index date. Patients who received the HD diagnosis before the first date of colchicine use or the year of index date before 2000 were excluded. Meanwhile, all the patients with hypertension, diabetes, and hyperlipidemia have received the medication for these comorbidities after index date. The criteria for these medications usage were in accordance with Taiwan guidelines.

### Propensity Score-Matching Approach

We used a propensity score-matching approach to establish a matched comparison cohort by balancing covariates between colchicine users and non-users to minimize selection bias. The propensity score was computed based on age, sex, medication, comorbidities and index date, the presence of comorbidities such as liver disease, pulmonary embolism, diabetes, hypertension, hyperlipidemia, chronic kidney disease, coronary artery disease, stroke, gout, venous thrombosis, COPD, and medication use including the acetylcysteine, inhaled corticosteroids, oral steroids, non-steroid anti-inflammatory drugs, cyclophosphamide (CYC), azathioprine (AZA), and methotrexate (MTX). The diagnostic accuracy of comorbidities and medication based on ICD-9 codes had been examined in previous studies (Supplementary Table 1, ICD-9CM and the anatomical therapeutic chemical codes, ATC).

### Study Outcome

#### Pericarditis, Myocarditis, Endocarditis, Cardiomyopathy, Cardiac Arrhythmia, and Heart Failure Were Considered as a Group

As mention before, pathological-microbiological evidence, biological plausibility, animal models, and clinical evidence indicated the virus related with—pericarditis, myocarditis, endocarditis, cardiomyopathy, cardiac arrhythmia, and heart failure could be lumped as a group. Meanwhile, from the biological plausibility, colchicine has effects on the HD (10, 17, 28). These disorders are combined into one category of HD (*ICD-9-CM* codes 420–428) (29, 30). The relationship between the virus infection such as hepatitis B and coronary artery disease (31) (CAD) is controversial (31). Therefore, the CAD did not enter into the HD in this study.

The outcome of interest was the occurrence of HD, which was defined as the first diagnosis of HD (*ICD-9-CM* 420-428). Patients were censored at the earliest date of HD diagnosis, insurance withdrawal with reason such as loss of insurance eligibility, death, or the end of the study period.

#### Statistical Analysis

The distributions of demographics, baseline comorbidities, and baseline medication status were compared between the colchicine users and the comparison cohort in both the unmatched and matched cohorts. We used the chi-square test to determine differences in age groups (18–49, 50–64, and > 65 years), sex, comorbidity, and medication between the cohorts. Continuous age distribution is presented as medians with interquartile ranges (*IQRs*) and examined using the Mann–Whitney *U*-test. The incidence rate of HD was calculated as the number of events divided by the sum of person-years (per 1,000 person-years) for each cohort.

#### Time-Dependent Analysis

Because the frequency of colchicine use in the case cohort was dynamic, the status of colchicine use was measured every 6 months. We set colchicine as a time-dependent covariate in the Cox proportional hazards regression model to estimate the risk of HD as hazard ratios (*HRs*) and their 95% confidence intervals (*CIs*) to reduce bias of drug effect. Multivariate models were adjusted for age, sex, comorbidities, medications, and index date. The stratified analysis was examined for the risk of HD by the subgroups of age, sex, comorbidities medications, and index date. Furthermore, we evaluated the effect of duration of colchicine use (2–7, 8–30, 31–150, > 150 days) on the risk of HD. Differences in the cumulative incidence of HD between the cohorts were estimated using the Kaplan–Meier method with log rank test. The SAS (Version 9.4, SAS Institute Inc., Cary, NC, United States) was used for all data analyses. The two-sided significance level was set at *p* < 0.05.

#### Validation of the Myocarditis/Pericarditis/Endocarditis/Myopathy and Interstitial Lung Disease

The diagnosis of acute myocarditis was identified in the NHIRD based on the *ICD-9-CM* code 422. To evaluate the accuracy of this diagnosis, a validation study was performed to review the medical records of hospitalized patients with the *ICD-9-CM* code of 422. In Chang et al. study the confirmation examinations included serum virus marker detection, cardiovascular magnetic resonance imaging (MRI), and endomyocardial biopsy (EMB), the positive predictive value was 96.5% for myocarditis (32).

In Taiwan, the diagnosis of constrictive pericarditis was established on the basis of the findings of Doppler echocardiography (thickened or calcified pericardium), cardiac catheterization (elevated end-diastolic pressure and the “square root sign” of right ventricular pressure tracing), and pericardial biopsy. However, on the basis of the high positive predictive value (PPV) observed in the examination of ILD pericarditis (33). The *ICD-9-CM* code of pericarditis was widely used for its diagnosis in the NHIRD (34).

Li et al. reported that the specificity and sensitivity of cardiac sonography with B-type natriuretic peptide (BNP) levels in detecting cardiomyopathy were 81.25% and of 70.00%, respectively (35). In Taiwan, the coding of the ICD-9CM for cardiomyopathy was based on the same criteria as those used in the previous study. In the ILD cohort with respiratory failure, which is listed as a catastrophic illness, the diagnosis of the respiratory failure-related diseases such as heart failure based on the *ICD-9-CM* were strict (14, 32, 36). In a previous study, the insurance claims data of Taiwan had a 92% accuracy for patients with ≥ 1 hospitalization in a year (13). The diagnostic accuracy of cardiac arrhythmia and heart failure based on the ICD-9-CM code in Taiwan NHIRD has been confirmed in previous reports also (37). Altogether, the diagnosis of HD based on the ICD-9CM is acceptable.

## Results

Initially, patients with ILD were identified including 16,867 colchicine users and 65,073 non-users (included in the unmatched cohort). After propensity score matching, 12,253 colchicine users and 12,253 non-users were included in the matched cohorts. Before matching, the patients in the colchicine cohort were older, were predominantly men, and had a higher proportion of comorbidities and medication use (*p* < 0.001). In the matched cohort, most variables were evenly distributed between the colchicine cohort and colchicine non-users. The median ages of the colchicine users and non-users were 51.6 (*IQR* = 41.8–61.4) years and 52.0 (*IQR* = 42.2–61.6) years, respectively. Both the cohorts included more women than men. Comorbidity and medication were balanced between the cohorts except for liver disease (*p* = 0.02), diabetes (*p* = 0.03), and steroid use, anti-inflammatory disease, and immunosuppressant (*p* = 0.001, Table 2).

**TABLE 2:** Demographic characteristics and comorbidities in the propensity-score-matched cohorts with and without colchicine used among patients with interstitial lung disease with viral infection.

	No matched			Propensity score matched		
			
	Colchicine			Colchicine		
			
	No	Yes		No	Yes	
					
Variable	*N* = 65,073	*N* = 16,867	*p*-value	*N* = 12,253	*N* = 12,253	*p*-value
Age, year			<0.001			0.02
18–49	34,666 (53.3)	7,752 (46.0)		5,323 (43.4)	5,523 (45.1)	
50–64	20,781 (31.9)	5,894 (34.9)		4,675 (38.2)	4,472 (36.5)	
65 +	9,626 (14.8)	3,221 (19.1)		2,255 (18.4)	2,258 (18.4)	
Median ± (IQR)^†^	48.8 (37.0–58.8)	51.4 (41.6–61.7)	<0.001	52.0 (42.2–61.6)	51.6 (41.8–61.4)	0.08
Sex			<0.001			0.001
Female	37,161 (57.1)	5,358 (31.8)		5,543 (45.2)	5,031 (41.1)	
Male	27,912 (42.9)	11,509 (68.2)		6,710 (54.8)	7,222 (58.9)	
**Comorbidity**						
Liver disease	24,974 (38.4)	8,558 (50.7)	<0.001	6,324 (51.6)	6,147 (50.2)	0.02
Pulmonary embolism	339 (0.52)	141 (0.84)	<0.001	80 (0.65)	83 (0.68)	0.81
Diabetes	8,530 (13.1)	3,056 (18.1)	<0.001	2,230 (18.2)	2,099 (17.1)	0.03
Hypertension	30,840 (47.4)	11,144 (66.1)	<0.001	7,758 (63.3)	7,648 (62.4)	0.15
Hyperlipidemia	40,605 (62.4)	13,224 (78.4)	<0.001	9,632 (78.6)	9,514 (77.7)	0.07
Chronic kidney disease	3,418 (5.25)	2,113 (12.5)	<0.001	1,149 (9.38)	1,141 (9.31)	0.86
CAD	13,821 (21.2)	5,024 (29.8)	<0.001	3,689 (30.1)	3,630 (29.6)	0.41
Stroke	3,533 (5.43)	1,360 (8.06)	<0.001	893 (7.29)	828 (6.76)	0.10
Gout	7,945 (12.2)	11,765 (70.0)	<0.001	7,151 (58.4)	7,151 (58.4)	0.99
Venous thromboembolism	1,795 (2.76)	670 (3.97)	<0.001	409 (3.34)	406(3.31)	0.91
COPD	3,560 (5.47)	1,379 (8.18)	<0.001	953 (7.78)	9,02 (7.36)	0.22
**Medications**						
Acetylcysteine	5,252 (8.07)	1,728(10.2)	<0.001	1,170 (9.55)	1,182 (9.65)	0.79
Inhaled corticosteroids (ICSs)	2,271 (3.49)	779 (4.62)	<0.001	519 (4.24)	502 (4.10)	0.59
Other medications	26,836 (41.2)	10,073 (59.7)	<0.001	6,744 (55.0)	6,276 (51.2)	0.001

*Chi-square test;*

*^†^Mann-Whitney test. Liver disease: including liver cirrhosis, hepatitis B, hepatitis C.*

*CAD, coronary artery disease; COPD, chronic obstructive pulmonary disease.*

*Other medications: oral steroids, statins, anticoagulants-warfarin, aspirin, and clopidogrel, anti-inflammatory drugs-Non-steroid anti-inflammatory drugs (NSAIDs), immunosuppressant’s-cyclophosphamide (CYC), azathioprine (AZA), Methotrexate (MTX).*

At the end of the follow-up period, the risk of HDs was significantly lower in the colchicine users than in the non-users both in the unmatched and matched cohorts. In the unmatched cohort, the median follow-up period was 7.08 (*IQR* = 3.82–10.4) years and 6.55 (*IQR* = 3.39–10.1) years for the colchicine users and colchicine non-users, respectively. In the matched cohort, the median follow-up period was 7.17 (*IQR* = 3.87–10.5) years and 6.98 (*IQR* = 3.68–10.4) years for the colchicine users and non-users, respectively. Multivariable time-dependent Cox regression analysis indicated that the incidence rate of HD was significantly lower in the colchicine users than in the non-users (unmatched cohort: *aHR* = 0.86, 95% CI = 0.81–0.90; matched cohort: *aHR* = 0.87, 95% CI = 0.82–0.92, Table 3).

**TABLE 3:** Incidence and HRs of HD in colchicine users compared with those in colchicine non-users by Cox proportional hazard models with time-dependent exposure covariates.

	No matched		Propensity score matched	
		
	Colchicine		Colchicine	
		
	No	Yes	No	Yes
Variable	(*N* = 65,073)	(*N* = 16,867)	(*N* = 12,253)	(*N* = 12,253)
Person-years	442,452	120,883	86,824	88,671
Follow-up time (y), Median ± (IQR)	6.55 (3.39–10.1)	7.08 (3.82–10.4)	6.98 (3.68–10.4)	7.17 (3.87–10.5)
**Heart diseases**				
Event	9,842	2,555	2,101	1,833
Rate^#^	22.2	21.1	24.2	20.7
Crude HR (95% CI)	1 (Reference)	0.96 (0.92, 1.00)*	1 (Reference)	0.86 (0.80, 0.91)***
Adjusted HR^†^ (95% CI)	1 (Reference)	0.86 (0.81, 0.90)***	1 (Reference)	0.87 (0.82, 0.92)***

*Rate#, incidence rate, per 1,000 person-years; Crude HR, relative; Adjusted HR^†^multivariable analysis including age, sex, comorbidities and medications.*

*p < 0.05; ***P < 0.001.

After stratification by age (18–49, 50–64, and > 65 years old), sex, and comorbidities, we observed a significantly lower risk of HD in the colchicine cohort in several subgroups. In the unmatched cohort, the adjusted HR for risk of HD was significantly lower in the colchicine users in all the age groups (*p* < 0.001), both male and female patients (*p* < 0.001), and patients with comorbidities (*p* < 0.001). In the matched cohort, a significant association between colchicine and a decreased risk of HD was observed in the 18–49- and 50–64-year-old age groups (*p* < 0.001), both female (*p* < 0.001) and male (*p* < 0.05) patient groups, and patients with comorbidities (*p* < 0.001, Table 1).

The association between the duration of colchicine use and the risk of HD was analyzed. Compared with the colchicine non-users, the patients who were prescribed colchicine for 2–7 days (*aHR* = 0.89, 95% CI = 0.81–0.98), 8–30 days (*aHR* = 0.84, 95% CI = 0.76–0.94), 31–150 days (*aHR* = 0.90, 95% CI = 0.80–0.99), and > 150 days (*aHR* = 0.83, 95% CI = 0.74–0.93) had a significantly lower risk of HD (Table 4).

**TABLE 4:** Incidence and adjusted HR of HD stratified by duration of colchicine therapy in patients with interstitial lung disease with virus infection cohort in the propensity-score-matched cohort.

Medication exposed	*N*	Event	Person-year	Rate	^a^Adjusted HR (95% CI)^†^
Non-colchicine Colchicine*	12,253	2,101	86,824	24.2	1.00
2–7 days	4,423	653	31,868	20.5	0.89 (0.81, 0.98)*
8–30 days^#^	2,855	416	20,600	20.2	0.84 (0.76, 0.94)**
31–150 days	2,612	407	18,697	21.8	0.90 (0.80, 0.99)*
>150 days	2,363	357	17,506	20.4	0.83 (0.74, 0.93)**

*^#^The cumulative use day are partitioned in to 4 segments by quartile.*

*^a^Adjusted HR^†^: multivariable analysis including age, sex, comorbidities, and medications.*

*p < 0.05, **p < 0.01.

**FIGURE 3:**
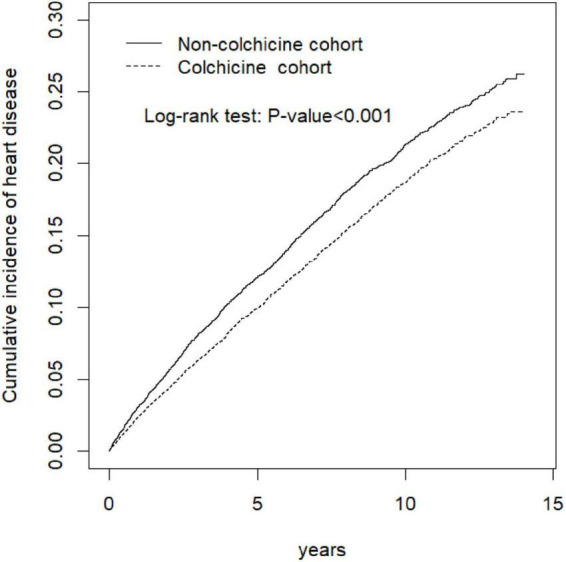
Cumulative incidence of heart disease curves for colchicine users and colchicine non-users by propensity score matched.

The results of Kaplan–Meier analysis revealed that the colchicine cohort exhibited a significantly lower cumulative risk of HD in subsequent years compared with the colchicine non-users (log-rank *p* < 0.001, Figure 3).

## Discussion

This study examined the relationship between colchicine use and the incidence of HD in ILD with viral infection. This is the first study to demonstrate the relationship between colchicine use and HD risk in ILD with a viral infection. The results of this study indicated that colchicine with 2–7 or 8–30 days use was associated with a lower risk of HD. In Deftereos et al. report revealed that the patients having catheter ablation of atrial fibrillation (AF) with 3 days of colchicine use, these colchicine users with lower levels of C-reactive protein (CRP) and IL-6 had a lower incidence of cardiac arrhythmia, such as AF recurrence, than did colchicine non-users; this finding is in accordance with that of the present study also (38).

A critical finding of this study is that the medium (31–150 days) and long-term use (> 150 days) of colchicine were associated with a lower risk of HD also. Fiolet et al. reported the levels of CRP, an inflammatory marker, decreased after 1 month of colchicine treatment, and the MRI findings of myocarditis revealed improvement, thus supporting this finding (39). Similar to meta-analysis of Kofler et al. report, the use of colchicine for > 150 days resulted in the lowest risk of HD (40). Thus, colchicine can be used for a long term without drug resistance in HD. This finding indicates that colchicine has a potential role in the auxiliary prevention of HD.

Another crucial finding is that patients with ILD with comorbidities had a lower risk of HD. One explanation is that these patients with comorbidities such as CAD or stroke had a shorter lifespan; thus, they did not have adequate time to develop the risk of HD. Another explanation is that those patients with multiple morbidities have higher medical services under multiple disciplinary team, leading to higher adherence of anti-hypertension drugs and optimal lifestyle modifications (41). Thus, these integrative policy contributing to the lower *aHR* for HD (42). These speculations merit further research.

The inappropriate prescription of colchicine results in side effects, especially in older patients, and thus affects the optimal duration for the prevention of HD (43–45). Moreover, older patients have a higher frequency of hypertension, liver cirrhosis, and diabetes, which are predisposing factors for HD. The adherence rate of the anti-hypertension drug for elderly is only about 57.6% (46). Thus, in this study, the suboptimal adherence of colchicine and anti-hypertension drugs may explain the no significant effect of colchicine use on HD in the older patients.

It is interesting to note that both the short-term or long-term use of colchicine exerted a potential effect on HD in ILD cohort, this result is similar to recent virus infection-related study (11, 47, 48). Moreover, Deftereos et al. performed a randomized clinical trial of patients with coronavirus infection and observed that participants who received colchicine had significantly improved time to clinical deterioration with HD (48). These recent study reported the benefit of colchicine in the prevention of coronavirus-related heart injury (11, 48, 49).

However, many other confounding factors may affect the *aHR* for HD. For example: (1) fetal viral infection with respiratory failure may result into short-life span which was not inadequate to get HD; (2) viral infection with comorbidities may contribute to high frequency medical services with multiple disciplinary services, leading to higher adherence of medications for prevention of HD. These two scenarios may contribute to lower *aHR* for HD also. Thus, our results could not indicate that colchicine can attenuate the *aHR* through the eradication of the viral infection.

In summary, these results imply that colchicine may play an auxiliary role in attenuating the risk of virus-related heart injury, especially in gout or non-gout diseases such as CAD, stroke, hypertension, hyperlipidemia, venous thrombosis, pulmonary embolism, and liver cirrhosis regardless of duration of use. Intriguingly, our data also allude to potential benefits of short-term colchicine use in preventing incident HD in this predisposed population. Owing to this retrospective study, these findings warrant further study.

### Strength

First, ours is the first large-scale study to investigate the effect of colchicine use on HD risk among ILD. Second, propensity-score matching ensured robust internal validity. Third, comorbidities, such as diabetes, hypertension, hyperlipidemia, gout, venous thrombosis, and pulmonary embolism, were examined in this study instead of lifestyle factors; stroke was used to represent environmental and economic status; cirrhosis, AF, chronic kidney disease, and CAD represented other HDs in ILD. Fourth, anti-inflammatory drugs and immunosuppressants were evaluated in the analysis to avoid confounding factors in the management of ILD and IPF. For example, the effect of the immunosuppressant such as steroids has aggravated the risk of the HD among the ILD cohort. Fifth, anti-IL-6 is expensive, whereas colchicine is cheap. Because colchicine can attenuate cytokine storm observed in patients with Coronavirus infection by exerting anti-inflammatory effects through the same pathway as that noted in anti-IL-6, thus, if the ILD patients concurrent with virus infection having the colchicine use, these groups with comorbidities may continue to receive the colchicine use in the Coronavirus era (50).

### Limitation

This study has several limitations. First, the NHIRD does not contain biochemical and clinical data, such as CRP, IL, PaO_2_/FiO_2_, glucose, SBP, DBP, MBP, and lifestyle data, such as smoking status. Second, some patients may not have received their prescribed medication or the prescribed dose, leading to exposure misclassification. Third, although our analysis included a wide range of potential confounding factors, our observational study still had potential residual confounders and indication bias. Fourth, the diagnosis of myocarditis based on clinical symptoms alone is unreliable, and subclinical myocarditis may escape clinical early detection. Therefore, myocarditis can be underdiagnosed in the clinical setting. Fifth, the minimal criteria to diagnose idiopathic cardiomyopathy include left ventricular diastolic dysfunction or reduced left ventricular ejection fraction, pathological left ventricular hypertrophy, and interstitial fibrosis. The potential cardiovascular risk factors for cardiomyopathy such as ischemic HD (IHD), rheumatic HD (RHD), valvular HD (VHD), congenital HD (CHD), stroke, and hyperlipidemia were retrieved from ambulatory care and inpatient claims between index date and date of end-of-follow-up and considered as potential confounders. After ruling out acquired disorders such as IHD, RHD, VHD, and CHD, the clinician could identify the cardiomyopathy based on the findings of a non-invasive echocardiogram, such as N-terminal pro-B-type natriuretic peptide (BNP) levels with LV end-diastolic maximum wall thickness (51). However, the NHI claims provided no information about detailed medical records and investigation results.

Finally, the completing risk, such as that of stroke or CAD or cancer in older patients, may be a confounding factor leading to comorbidities with lower *aHR* for HD.

## Conclusion

The addition of short-term or long-term colchicine to standard medical therapy may have benefit to prevent the HD among the ILD patients concurrent with virus infection or comorbidities even in elderly patients.

## Data Availability Statement

The datasets presented in this article are not readily available because the dataset used in this study is held by the Taiwan Ministry of Health and Welfare (MOHW). The Ministry of Health and Welfare must approve our application to access this data. Any researcher interested in accessing this dataset can submit an application form to the Ministry of Health and Welfare requesting access. Requests to access the datasets should be directed to stcarolwu@mohw.gov.tw.

## Ethics Statement

This study was approved by the Research Ethics Committee of China Medical University Hospital (CMUH104-REC2-115-AR-4). Written informed consent for participation was not required for this study in accordance with the national legislation and the institutional requirements.

## Author Contributions

J-JY and T-WH: conception and design. C-HK: administrative support. All authors: collection and assembly of data, data analysis and interpretation, manuscript writing, final approval of manuscript, contributed significantly, and that all authors are in agreement with the content of the manuscript.

## Conflict of Interest

The authors declare that the research was conducted in the absence of any commercial or financial relationships that could be construed as a potential conflict of interest.

## Publisher’s Note

All claims expressed in this article are solely those of the authors and do not necessarily represent those of their affiliated organizations, or those of the publisher, the editors and the reviewers. Any product that may be evaluated in this article, or claim that may be made by its manufacturer, is not guaranteed or endorsed by the publisher.

## Supplementary Material

Supplementary Figure 1 Speculations intertitial lung disease, virus, heard disease coxesit, and interplay with NLRP3 inflammasome.
