# Supplementary material for: Colchicine Is a Weapon for Managing the Heart Disease Among Interstitial Lung Disease With Viral Infection: Have We Found the Holy Grail?
Source: Front Cardiovasc Med. 2022 Jun 28;9:925211. doi: 10.3389/fcvm.2022.925211 (PMC9273766; doi:10.3389/fcvm.2022.925211)
Supplement: Supplementary file 1 [file Table_1.docx]

Supplemental Table 1. Full names of ICD-9 CM, comorbidities and anatomical therapeutic chemical codes.

| Autoimmune disease-ILD | | |
| --- | --- | --- |
| 135 | sarcoidosis | |
| 237.7 | neurofibromatosis | |
| 272.7 | lipidoses | |
| 277.3 | amyloidosis | |
| 277.8 | other specified disorders of metabolism (including eosinophilic granuloma) | |
| 446.21 | Goodpasture's syndrome | |
| 446.4 | Wegener's granulomatosis | |
| Environment, -ILD | | |
| 495 | extrinsic allergic alveolitis (EAA, HP) | |
| 500 | coal workers’ pneumoconiosis | |
| 501 | asbestosis | |
| 502 | pneumoconiosis due to other silica or silicates | |
| 503 | pneumoconiosis due to other inorganic dust | |
| 504 | pneumonopathy due to inhalation of other dust | |
| 505 | pneumoconiosis (unspecified) | |
| 506.4 | chronic respiratory conditions due to chemicals, gases, fumes, and vapors | |
| 508.1 | chronic and other pulmonary manifestations due to radiation | |
| 508.8 | respiratory conditions due to other specified external agents | |
| Pulmonary fibrosis, IPF with virus? | | |
| 515 Virus | | postinflammatory pulmonary fibrosis |
| 516 Virus | | other alveolar and parietoalveolar pneumonopathy, which includes |
| 516.30 | | idiopathic interstitial pneumonia not otherwise specified |
| 516.31 | | idiopathic pulmonary fibrosis (IPF) |
| 516.32 | | idiopathic nonspecific interstitial pneumonitis(iNSIP) |
| 516.33 | | acute interstitial pneumonitis |
| 516.34 | | respiratory bronchiolitis interstitial lung disease |
| 516.35 | | idiopathic lymphoid interstitial pneumonia |
| 516.36 | | cryptogenic organizing pneumonia |
| 516.37 | | desquamative interstitial pneumonia |
| Connective tissue disease (CTD) -ILD | | |
| 517.2 | | lung involvement in systemic sclerosis |
| 517.8 | | lung involvement in other diseases classified elsewhere; |
| 518.3 | | pulmonary eosinophilia |
| 555.0-555.9 | | Crohn's disease - |
| 710 | | diffuse diseases of connective tissue |
| 710.0 | | lupus |
| 710.1 | | systemic sclerosis |
| 710.2 | | Sjögren’s disease |
| 710.3 | | dermatomyositis |
| 710.4 | | polymyositis |
| 714.81 | | rheumatoid lung |
| 720 | | ankylosing spondylitis and other inflammatory spondylopathies |
| 759.5 | | tuberous sclerosis |
| Comorbidities | | |
| 415.0,415.1 | | Pulmonary embolism |
| 415.11,415.19,451.11,451.19,451.81,453.40,453.41,453.42,4538–4539 | | Venous thrombosis |
| 362 | | Retinal disorders |
| 491,492,496 COPD | | Chronic obstructive pulmonary disease |
| 307.4  780.5 | | Sleep disorder, |
| 205 | | Diabetes |
| 401-405 | | Hypertension |
| 272 | | Hyperlipidemia |
| 291, 303, 305, 503.81  571.0- 571.3,  and 790.3 | | Alcohol-related illness |
| 585 | | Chronic kidney disease |
| 410-414 | | CAD |
| 430-438 | | Stroke |
| 140-208 | | Cancer |
| 274 | | Gout |
| 290-319 | | Mental disorder |
| Virus infection coexist with IPF | | |
| 042.0 | | Human immunodeficiency virus [HIV] disease |
| 053.0 | | Herpes virus |
| 070.20 070.22  070.30 070.32 | | **Hepatitis B** |
| 070.41 070.44  070.51 070.54 | | HepatitisC |
| 075.0 | | Epstein-Barr virus |
| 078.5 | | cytomegalic infection |
| 079.0 | | Adenovirus infection |
| 079.1 | | Echo virus infection |
| 079.2 | | Rhinovirus infection |
| 079.3 | | Human papillomavirus |
| 079.4 | | Retrovirus in conditions |
| 079.5 | | Respiratory syncytial virus (RSV) |
| 079.81 | | Hantavirus infection |
| [079.82](http://www.icd9data.com/2012/Volume1/001-139/070-079/079/079.81.htm) | | SARS-associated coronavirus |
| 079.83 | | Parvovirus B19 |
| 079.88 | | Other specified chlamydial infection |
| 079.89 | | Unspecified viral infections |
| 480 | | Virus pneumonia |
| 486 | | Pneumonia (including bacteria) |
| 487-488 | | Influenza |

| ICD-9CM | Full name |
| --- | --- |
| 401-405 | hypertension |
| 410–414 | coronary artery disease |
| 420 | acute pericarditis |
| 421 | endocarditis |
| 422 | myocarditis |
| 423 | other disease of pericardium |
| 424 | other disease of endocardium |
| 425 | cardiomyopathy |
| 426 | conduction disorder |
| 427 | cardiac arrhythmia |
| 428 | heart failure |
| 272 | dyslipidemia |
| 251.0, 251.2 | hypoglycemia |
| 278.00 | obesity |
| 274 | gout |
| Liver disease |  |
| 571 | liver cirrhosis |
| 070.2, 070.3 | hepatitis B |
| 070.44,070.54 | hepatitis C |
| 486 | pneumonia |
| 712 | **c**rystal arthropathies |
| 135 | sarcoidosis |
| 136.1 | Behcet's syndrome |
| 279.49 | autoimmune disease |
| 696.0, 696.1, 696.8 | psoriasis |
| ATC code | |
| B01AA03 | warfarin |
| B01AC06 | aspirin |
| B01AC04 | clopidogrel |
| C10AA | statins |
| M01A | NSAIDs |
| L01AA01 | cyclophosphamide(CYC) |
| L04AX01 | azathioprine(AZA) |
| L04AX03 | methotrexate(MTX) |
| R05CB01 | acetylcysteine |
| H02AB | inhaled corticosteroids |
| R03BA | oral steroids |
| M01A | nonsteroid anti-inflammatory drugs |
| C09AA  C09CA  C08  C07A  C03 | angiotensin converting  enzyme inhibitors  angiotensin-2 receptors blockers  calcium channel blockers  beta blockers  diuretics |
