# Supplementary material for: Colchicine Is a Weapon for Managing the Heart Disease Among Interstitial Lung Disease With Viral Infection: Have We Found the Holy Grail?
Source: Front Cardiovasc Med. 2022 Jun 28;9:925211. doi: 10.3389/fcvm.2022.925211 (PMC9273766; doi:10.3389/fcvm.2022.925211)
Supplement: Supplementary file 2 [file Table_2.docx]

**Supplemental Table 2.**

Corresponding table for biomarker, Charlson Comorbidity Index.

Comorbidities, Medications in ILD cohort.

| **Biomarker** | **Charlson Comorbidity Index** | **Comorbidities, medications** |
| --- | --- | --- |
| CRP  IL | AIDS | Immunosupprasants  Steroids |
| CRP  IL | Metastatic solid tumor  Any tumor  Lymphoma  Leukemia | Gout  Hypertension  Hyperlipidemia  DM |
| CRP  IL | Moderate or severe liver disease  Mild liver disease  Ulcer disease | Liver disease |
| CRP  IL  Glucose | Diabetes with end organ damage  Diabetes | Diabetes |
| SBP  DBP  MBP | Moderate or severe renal disease | Chronic kidney  disease |
| SBP  DBP  MBP | Hemiplegia  Cerebrovascular disease  Dementia | Stroke  Hypertension |
| PaO2/FiO2 | Chronic pulmonary disease | COPD  ICS |
| PaO2/FiO2 | Peripheral vascular disease | Pulmonary embolism  Venous thrombosis |
| PaO2/FiO2 | Connective tissue disease | Acetylcysteine |
| SBP  DBP  MBP  PaO2/FiO2 | Congestive heart failure | hypertension |
