# Supplementary figures and images for: Colchicine Is a Weapon for Managing the Heart Disease Among Interstitial Lung Disease With Viral Infection: Have We Found the Holy Grail?
Source: Front Cardiovasc Med. 2022 Jun 28;9:925211. doi: 10.3389/fcvm.2022.925211 (PMC9273766; doi:10.3389/fcvm.2022.925211)

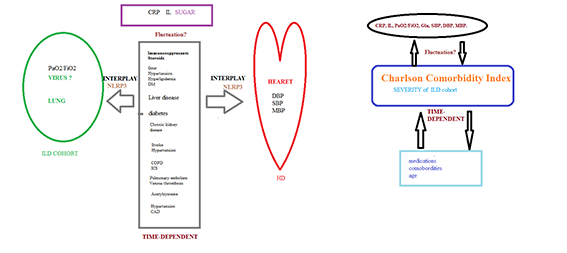

Supplement: Supplementary Figure 1 — Speculations intertitial lung disease, virus, heard disease coxesit, and interplay with NLRP3 inflammasome. [file Image_1.tif]
